# Community engagement strategy for increased uptake of routine immunization and select perinatal services in north-west Ethiopia: A descriptive analysis

**DOI:** 10.1371/journal.pone.0237319

**Published:** 2020-10-29

**Authors:** Shiferaw Dechasa Demissie, Naoko Kozuki, Comfort Z. Olorunsaiye, Petros Gebrekirstos, Siraj Mohammed, Lilian Kiapi, Tracey Chantler, Emilie Karafillakis, Justine Landegger

**Affiliations:** 1 International Rescue Committee, Addis Ababa, Ethiopia; 2 International Rescue Committee, New York City, NY, United States of America; 3 International Rescue Committee, London, United Kingdom; 4 London School of Hygiene & Tropical Medicine, London, United Kingdom; IAVI, UNITED STATES

## Abstract

**Background:**

Routine immunization coverage has stagnated over the past decade and fallen short of WHO targets in Ethiopia. Community engagement strategies that reach beyond traditional health systems may reduce dropout and increase coverage. This evaluation assesses changes in immunization, postpartum family planning, and antenatal care coverage after implementation of an enhanced community engagement and defaulter tracing strategy, entitled “Fifth Child” project, across two districts in Benishangul-Gumuz Regional State (BGRS), Ethiopia.

**Methods and findings:**

A formative evaluation was conducted to examine the contribution of the strategy on immunization, postpartum family planning and antenatal care utilization in Assosa and Bambasi districts of BGRS. The quantitative findings are presented here. Routine and project-specific data were analyzed to assess changes in uptake of childhood vaccinations, postpartum family planning and antenatal care. Between January 2013 and December 2016, pentavalent-3 coverage increased from 63% to 84% in Assosa, and from 78% to 93% in Bambasi. Similarly, measles vaccine coverage increased from 77% to 81% in Assosa, and from 59% to 86% in Bambasi. Approximately 54% of all eligible infants across both *woredas* defaulted on scheduled vaccinations at least once during the period. Among defaulting children, 84% were identified and subsequently caught up on the vaccinations missed. Secondary outcomes of postpartum family planning and antenatal care also increased in both *woredas*.

**Conclusion:**

The “Fifth Child” project likely contributed to enhanced immunization performance and increased utilization of immunization and select perinatal health services in two *woredas* of BGRS. Further research is required in order to determine the impact of this community engagement strategy.

## Background

Immunization is one of the most cost-effective means of preventing childhood illnesses and annually averts 2–3 million deaths from vaccine-preventable diseases [1, 2]. With initiatives like the Expanded Program on Immunization (EPI), global immunization coverage has generally increased, reducing under-five mortality [3]. However, coverage has stagnated around 85% for several years [4]. Ethiopia’s national coverage of the third dose of pentavalent vaccine (diphtheria, tetanus, pertussis, Hemophilus influenza type B, hepatitis B) (Pentavalent-3) was 37% and the dropout rate between the first and third doses was 43% in 2011 [5]. The country is one of 35 WHO Member States that fell short of the 2015 Global Vaccine Action Plan’s (GVAP) goal of 90% national pentavalent-3 coverage [6]. In Benishangul Gumuz Regional State (BGRS), pentavalent-3 coverage was 42%, just slightly above the national average, and 13% of infants in the region received no vaccines at all [5].

### Problem

Previous studies in Ethiopia identified several barriers to immunization service utilization. Lower household income [7], low maternal education levels [8, 9], and poor use of maternal health services, including antenatal care (ANC) and delivery [7, 8, 10], and distance to a health facility [11] have been shown to be negatively associated with use and completion of routine immunization. Mothers’ lack of knowledge of schedules and benefits of immunization has also been associated with default from scheduled immunizations [7, 8]. In BGRS specifically, according to *Woreda* (district) Health Office experts, primary reasons for low immunization coverage included: misunderstanding of the schedule, poor healthcare-seeking behavior, lack of confidence in the health system, limited access to services and irregular outreach sessions, low capacity among health workers, especially related to defaulter tracing, and vaccine shortage, most often due to poor forecasting and interruptions in the cold chain system for vaccine storage [12].

Low vaccination coverage highlights the need for an intervention that better engages caregivers in immunization programming while also addressing critical health system gaps. Thus, to improve select maternal, neonatal and child health (MNCH) outcomes and immunization performance in BGRS, the International Rescue Committee (IRC) implemented a project entitled the “Fifth Child Project—Closing the Immunization Gap” (FCP); highlighting that, globally, one in every five children does not receive basic vaccinations [12].

### Implementation strategy

The FCP was informed by a theory of change (see S1 Fig) that incorporates three main inter-related causal pathways: i) enhanced engagement on immunization and select perinatal services, at household and community levels, ii) improved defaulter tracing, and iii) underlying immunization systems support, that, combined, will lead to increased immunization coverage. The theory of change provided a framework for evaluating the FCP. National development goals and targets were also integrated within the project performance monitoring plan.

### Intervention

Integrated interventions included the design and rollout of a cross-cutting caregiver engagement and community co-management strategy and the introduction of two complementary tools: a color-coded health calendar (CCHC) and defaulter tracing tool (DTT). The Health Extension Workers (HEW), or the community-based health worker cadre in Ethiopia, were also trained in family planning counselling and provision of short-term family planning methods and implants.

### Intended outcomes

The FCP was designed to prompt collaboration between HEWs, Health Development Army (HDAs), and community (referred to as *kebeles*) leaders to, each year, register, counsel, and track more than 5,000 pregnant women and their infants for increased uptake of immunization and perinatal services and reach the following outcomes:

1. 85% pentavalent-1, pentavalent-3, and measles vaccine coverage.
2. 70% of defaulting infants receive catch-up vaccination.
3. 35% of postpartum mothers accept a modern family planning (FP) method, after referral by immunization service providers.

## Methods

### Study design

We conducted an evaluation, consisting of quantitative and qualitative methods, to assess FCP contribution in improving utilization of immunization and related perinatal services in BGRS. This paper describes the intervention and reports quantitative findings; qualitative methods and findings are presented in a complementary paper [13].

### Setting

Located in northwest Ethiopia, BGRS borders Sudan and South Sudan. Bambasi and Assosa districts (referred to as *woredas* in Ethiopia), lowland areas, inhabited by predominantly Muslim agrarian communities, were selected for the intervention. The majority of the population has poor access to health services.

**Fig 1:**
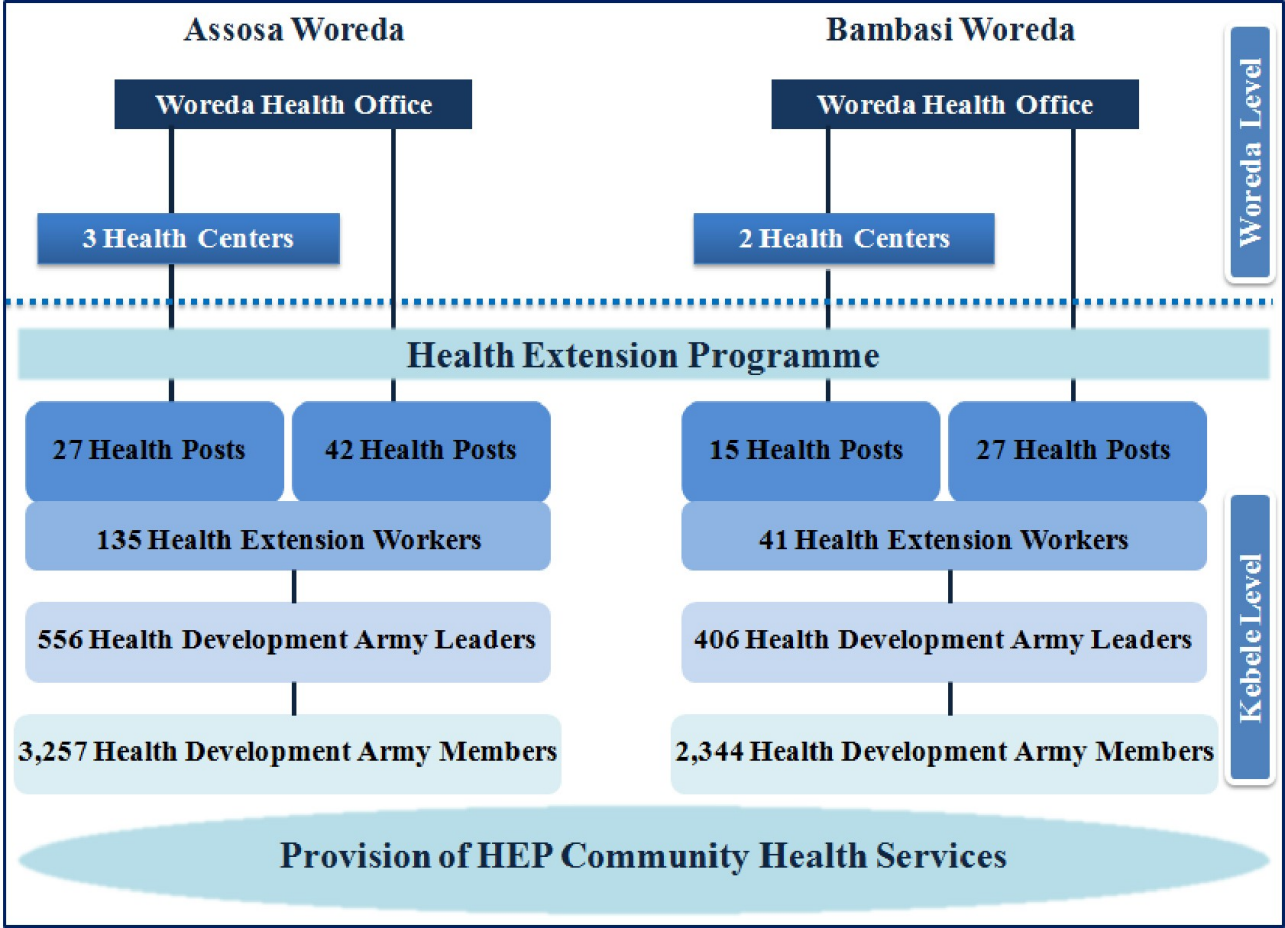
Distribution of health facilities and HEP personnel in Assosa and Bambasi.

The FCP was introduced in the context of Ethiopia’s Health Extension Program (HEP); rolled out in BGRS in 2009. The HEP serves as the primary vehicle for implementation of community-centered essential health packages and facilitates referral processes from grassroots to secondary and tertiary levels [14]. The lowest level of the referral system is the health post, usually one per village *(kebele)*, staffed by two salaried female health extension workers (HEWs) that provide preventive and basic curative services, including immunization, and conduct household visits and immunization outreach services in *kebeles*. Immunization is generally provided at fixed sites once or twice per week, or through monthly community-level outreaches. The government introduced a female-led health development army (HDA) in 2014 to extend the reach of the HEP and reinforce positive health practices. The HDA strategy creates a network of five households, each linked to an HDA model household, based on their adoption of health extension packages such as giving birth at a health facility, immunizing children, using modern family planning, and having latrine. The resident in the model household is designated as a member of the HDA and as the leader of those five households, also known as a 1-to-5 leader. Five 1-to-5 leaders are led by a 1-to-30 leader, referred to here as a Health Development Army Leader (HDAL), who in turn reports to HEWs. Health Extension Workers are either supervised by the *Woreda* Health Office or the nearest health center (Fig 1). HDAs and HDALs do not have formal required qualifications. The HDAs and HDALs are all volunteers and work part time with no pay per government policy.

### Implementation

The FCP was iteratively developed and implemented in all 114 *kebeles* across Assosa and Bambasi *woredas* between 2014 and 2016. Key tools, designed in consultation with the Regional Health Bureau and *Woreda* Health Offices, included a CCHC, called *Enat Mastawesha*, or ‘mother’s reminder’ in Amharic language, and a DTT. The project team hypothesized that the use of these tools within a larger community engagement strategy–that directly involves caregivers and specifically connects with existing community leadership structures–would address some identified barriers to service uptake.

The intervention had four components:

- The *Enat Mastawesha*, distributed to all households with pregnant women or infants, aimed to improve understanding of the timing and purpose of critical MNCH services. HEWs registered pregnant women as early as possible and HEWs distributed calendars during home visits, describing color-coded images and placing color-coded stickers on appointment dates to help remind families when to visit facilities. HDAs and HDALs conducted subsequent household visits to provide health education, appointment reminders, defaulter tracing and to mobilize households for health promotion activities. The IRC trained HEWs directly and facilitated HEW-led trainings for HDAs and HDALs on improving interpersonal communication skills for effective, tailored, counseling sessions. The intention was to increase the quality of home visits by making them more interactive and motivational, and support families to develop a plan for preventative healthcare-seeking. See Box 1.
- The DTT is a simple carbon-copy registration form used at the health post to record, by village, basic infant/caregiver information and vaccines missed based on the immunization registration book of the health post from where the list of defaulter children are identified. The number of defaulters were discussed using the DTT during pre-existing bi-monthly *kebele* command post meetings as a way of monitoring immunization performance. These meetings comprised representatives from different sectors (teachers, *kebele* leaders, HEWs, HDALs, representatives of youth and women groups) to review and follow-up on performance of development activities, including health services and planning corrective actions. During the *kebele* command post meetings, HEWs gave copies of the DTT to HDALs, who in turn informed HDAs about which defaulters needed to be visited and followed up for catch-up vaccinations. The HDAs then conducted home visits to counsel families whose children defaulted, or were never immunized, to promote catch-up vaccinations. After household visits were conducted, HDAs reported back to HDALs, who in turn updated the DTT and returned same to HEWs at the health post.
- Building upon existing *kebele* administrative structures, this intervention aimed to foster ownership and ongoing feedback between communities and health posts related to immunization services. *Kebele* leaders (and often HDAs as well) were informed by the HEWs and HDALs of any defaulters and participated in mobilizing caregivers to attend immunization sessions at the health post or during outreaches. In the hardest-to-reach places, *kebele* leaders attended outreaches to monitor whether: the sessions took place as planned, EPI supplies and vaccines were available, and defaulters attended for catch-up vaccinations. Similarly, HEWs and HDALs also discussed challenges encountered with *kebele* leaders and worked with command post members to jointly identify and plan corrective actions. Furthermore, monitoring observations were also discussed at monthly health facility meetings. Serious or unresolved issues from health facility or *kebele* meetings were presented at quarterly *woreda* meetings, also attended by *Woreda* Health Officers and *kebele* leaders, for further discussion and resolution.
- Basic health system support, addressing key service delivery gaps within the HEP, served as the foundation upon which to introduce tools and test the FCP approach. Underlying activities included: basic cold chain maintenance, vaccine transport, support for outreaches (e.g. fuel, per diems), and training and supervision of HEWs on EPI and FP, including implant insertion.

**Box 1. Household and caregiver engagement using the *Enat Mastawesha***

**Pregnancy period**: pregnant women and decision-makers in the home were visited and counseled by HEWs using the *Enat Mastawesha* and discussed ANC, birth planning and the importance of facility-based delivery.

**Immediate post-delivery period:** HEWs discussed and placed appointment stickers for postnatal, immunization and FP services with new mothers and their families; specifically alerting them about date/time of the next outreach. In case the child’s immunization schedule crossed into the subsequent calendar year, caregivers received an additional calendar for the subsequent year.

**Immunization dropout**: In case of missed immunizations, HEWs registered infants in the DTT and HDAs were informed to visit and counsel caregivers about catch-up immunization. During defaulter tracing visits, HDAs placed an additional sticker on the calendar to indicate the next outreach date. The HDA would revisit the same household a few days prior to the outreach to promote attendance, after which the HDAs informed their leaders to update and return the copy of the DTT to the HEWs.

### Program monitoring

Monthly program data on immunization and ANC were reviewed by the health program manager and the *Woreda* Health Officers. Additionally, during joint monthly supportive supervision visits conducted by the health manager and *Woreda* Health Officer, service delivery data and records were reviewed with the HEWs. During these visits, program staff also provided support and training to HEWs as needed.

Data on defaulter tracing and postpartum family planning that were not routinely collected in the government database were collated and checked for quality separately on a monthly basis by the project team under the supervision of the health manager.

The health manager updated the project database monthly and transmitted it to the country-level monitoring and evaluation (M&E) manager and country health technical unit. The country M&E manager and the technical team reviewed the data, analyzed and sent back feedback to the field manager, who in turn shared the feedback with the project team, Woreda Health Office and target health facilities. The M&E manager also maintained the database. The M&E manager conducted quarterly monitoring visits to the field office and *woreda* health offices to jointly review the data with the staff of the project and *woreda* health office.

### Timeline

**Fig 2:**
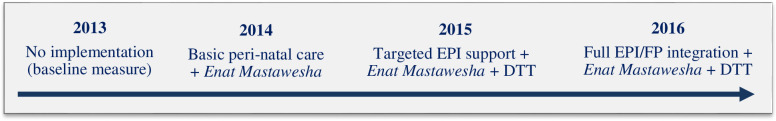
Phased introduction of “Fifth Child” intervention.

The timeline of the program rollout was in three phases as follows (see Fig 2): Phase 1 was the IRC’s general programme rollout (health workers trainings and mentorship, joint supportive supervision, quarterly review meetings and basic health system support) (year 2014, quarters 1 and 2); Phase 2 was after the rollout of the CCHC but before rollout of the DTT (year 2014, quarters 3 and 4, year 2015 quarters 1 and 2); and Phase 3 after the rollout of both interventions (year 2015 quarters 3 and 4, year 2016 quarters 1, 2, and 3).

### Variables

The outcome variables of interest were quarterly vaccinations administered and coverage rates, respectively, for pentavalent 1, pentavalent 3, and measles. The main exposure variable was the stage of intervention, coded as a categorical variable, with the reference category coded as prior to any intervention (year 2013), and other categories coded as Phases 1–3. The intervention was also coded as a binary variable comprising: before full implementation (reference category) and after full implementation (category 3 above). A confounding variable of interest was seasonality, coded as rainy season (July-Sep) versus the dry season (Oct-June.)

### Data sources

Immunization and ANC data were collected from the *woreda* routine Health Management Information System (HMIS) and stored in an Excel database on a password-protected computer. Some project data, not on the HMIS e.g. defaulters by type of vaccine and postpartum FP acceptors, were collected using a different database, deidentified and updated by the field-based health manager and sent to the IRC M&E Manager and health technical team for quality checks, collation, analysis and feedback.

### Analysis

Monthly immunization and ANC data were available from January 2013 to December 2016. Postpartum FP data were available from January-December 2016. The number of pentavalent-1, pentavalent-3, and measles vaccinations were aggregated into quarters (Jan-Mar, Apr-Jun, Jul-Sep, Oct-Dec) and analyzed. Immunization coverage with these vaccines was also calculated using the number of infants due for vaccination in each *woreda* (Table 1).

**Table 1:** Estimated number of infants (under one year of age) due for vaccination (2013–2016).

Year	Assosa		Bambasi	
	Annual	Monthly	Annual	Monthly
2013	3744	312	1976	165
2014	3738	312	2036	170
2015	3111	259	1891	158
2016	3237	270	2052	171

Multilevel linear regression analysis was conducted, with quarterly vaccinations administered and coverage rates as the dependent variable and the intervention stages as the independent variable. The regression controlled for seasonality and accounted for clustering at the *woreda* level. Because of the high correlation between calendar time and intervention, we could not statistically control for secular trends.

We also calculated the frequencies on variables related to defaulter tracing, postpartum FP, and ANC for the following indicators: 1) proportion of defaulters identified and subsequently immunized, 2) proportion of postpartum mothers who accepted a modern FP method, after referral by immunization service providers and 3) proportion of pregnant women who had one and four ANC visits, respectively. Modern FP methods include hormonal oral and injectable contraceptives, implants and intrauterine contraceptive devices.

Stata version 13 and Microsoft Excel were used for the analysis. P-value <0.05 was considered statistically significant.

### Ethics and consent

The evaluation was approved by the London School of Hygiene & Tropical Medicine Observational and Interventions Research Ethics Committee (Ref 10542) and the Regional Research Ethics Committee of BGRS Health Bureau. Aggregated immunization and ANC data were provided by the *Woreda* Health Office in deidentified form before use in this retrospective analysis. Aggregated, de-identified project data for postpartum FP and defaulter tracing were provided by the IRC Ethiopia Program Office.

## Results

This section presents FCP achievements based on routine project data. Fig 2 illustrates how the intervention evolved. Health Management Information System (HMIS) data from 2013 served as baseline prior to IRC implementation. In 2014, the *Enat Mastawesha* was introduced and MNCH activities commenced, supporting perinatal care, basic strengthening of EPI and FP services. Intensive EPI strengthening started in 2015 when the DTT was rolled out alongside related community co-management activities. By 2016, there was full implementation, including an integrated EPI/FP strategy.

### Primary outcomes

#### Immunization achievement and coverage

**Fig 3:**
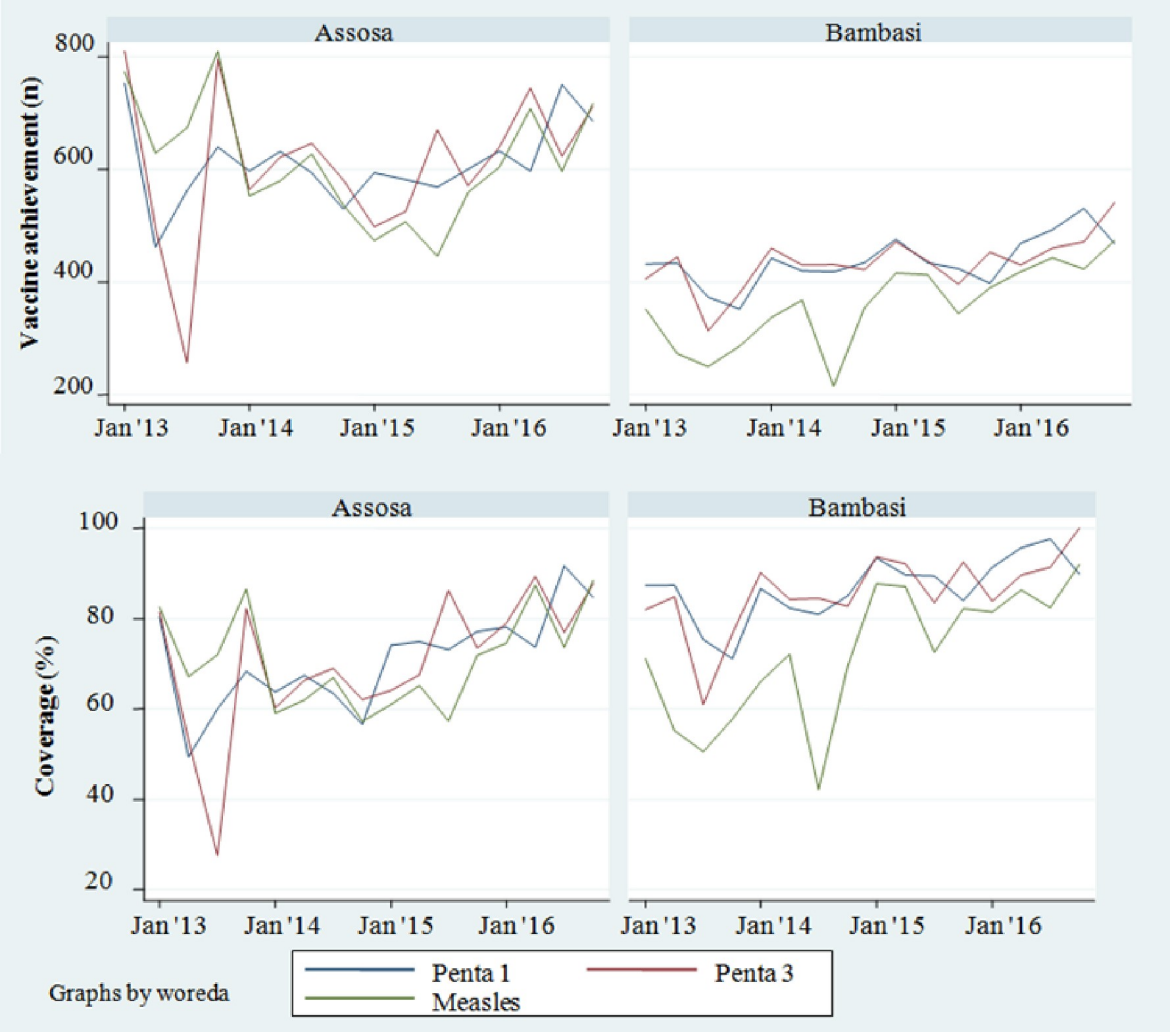
Quarterly vaccine achievement (number of vaccine doses administered) and coverage in Assosa and Bambasi, (Jan 2013-Dec 2016).

The trends of the quarterly vaccination achievement and the coverage show, in both *woredas*, an increase across the project period (Fig 3). Pentavalent-3 coverage increased from 67% in December 2013 to 84% by December 2016 in Assosa, and from 78% in December 2013 to 93% by December 2016 in Bambasi. Measles coverage increased from 77% to 81% in Assosa and from 59% to 86% in Bambasi during the same period.

#### Quarterly coverage of vaccination by intervention

There was a statistically significant increase in the coverage of pentavalent-1, pentavalent-3, and measles vaccines when the *Enat Mastawesha*, DDT and community co-management strategy were fully implemented (Table 2).

**Table 2:** Change in quarterly coverage (percentage points), by intervention (Jan 2013- Dec 2016).

	Pentavalent1		Pentavalent3		Measles	
	Beta	p	Beta	p	Beta	p
No IRC program	Ref					
+ IRC program implementation	2.2 (-6.5, 10.9)	0.616	4.2 (-7.4, 15.9)	0.478	-6.1 (-17.9, 5.7)	0.313
+ *Enat Mastawesha*	4.9 (-2.1, 11.8)	0.172	8.4 (-1.0, 17.7)	0.081	-0.7 (-10.3, 8.8)	0.879
+ *Enat Mastawesha*	13.3 (6.9, 20.0)	<0.001	18.4 (9.8, 26.9)	<0.001	12.4 (3.6, 21.1)	0.005
+defaulter tracing tool						
+community co-management						

Increases of similar magnitude were seen when comparing the quarters before and after full implementation (Table 3).

**Table 3:** Change in quarterly coverage (percentage points), comparing no implementation to full implementation (*Enat Mastawesha*, and defaulter tracing tool).

	Pentavalent1		Pentavalent3		Measles	
	Beta	p	Beta	p	Beta	p
Before full implementation	Ref					
After full implementation	10.9 (5.6, 16.2)	<0.001	14.2 (6.9, 21.4)	<0.001	13.8 (6.6, 20.9)	<0.001

#### Defaulter tracing

Out of 5,289 infants in both *woredas*, 2,830 (54%) defaulted on one or more scheduled vaccination visits between January-December 2016. This can include multiple counts for each child. Of the 2,830 defaulting children identified, 2,363 (84%) were successfully caught up on the missed vaccinations (results not shown in tables).

### Secondary outcomes

#### Uptake of postpartum family planning

**Fig 4:**
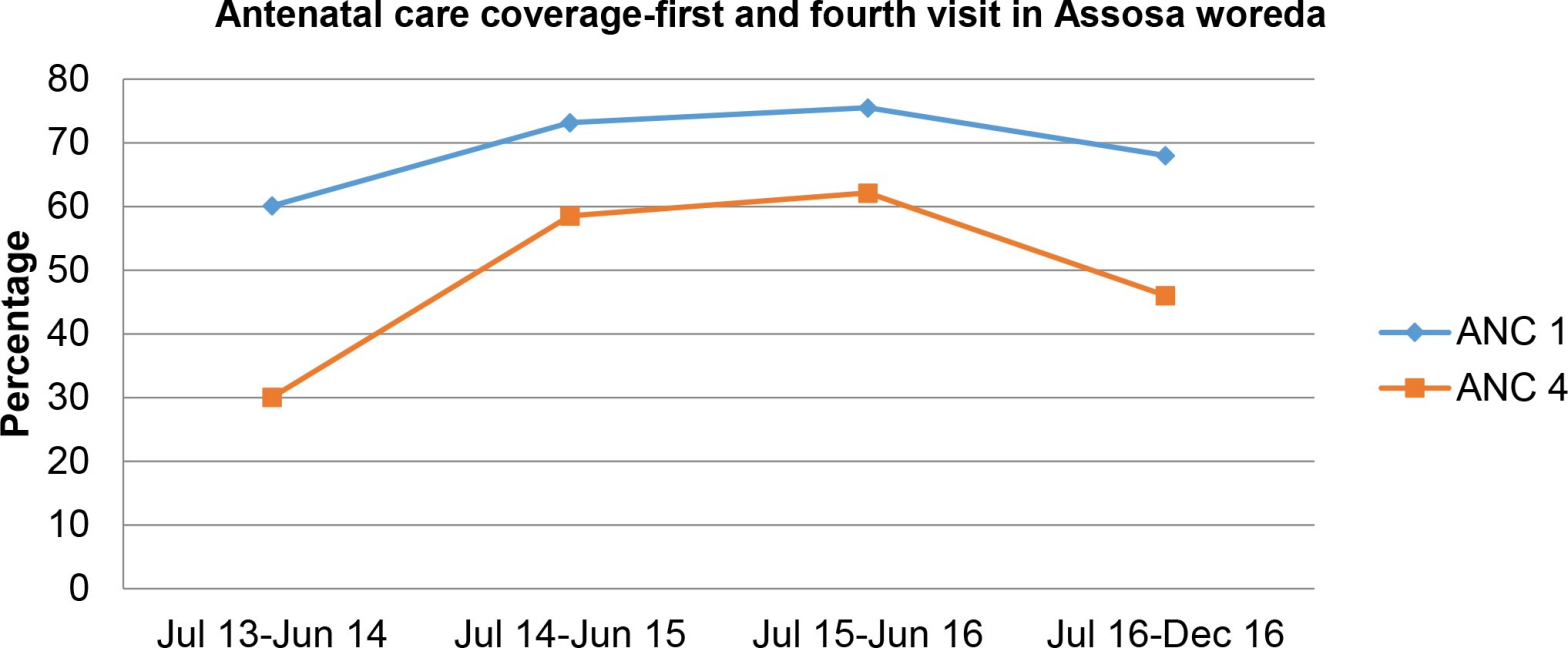
Performance of secondary outcomes during ‘Fifth Child’ intervention period.

The proportion of postpartum women adopting a modern FP method increased in both *woredas* from January-December 2016, starting at 12% and closing at 93% (of 1,752 deliveries) in Assosa and starting at 18% and closing at 62% (of 918 deliveries) in Bambasi (Fig 4).

#### Uptake of antenatal care

There was an overall increase in the uptake of ANC in both *woredas* (Fig 4). In Assosa, coverage of the first ANC visit initially increased from a baseline of 60% in June 2013 to 76% in June 2016, then declined to 68% by December 2016. Similarly, coverage of the fourth ANC visit increased from 30% in 2013 to 62% in June 2016, then declined to 46% by December 2016. Attendance at one and four ANC visits generally increased in Bambasi *woreda* from 67% to 95% and 26% to 42%, respectively, from June 2013 to December 2016.

## Discussion

This study assessed FCP performance by reviewing trends in uptake of routine immunizations, defaulter tracing, and select perinatal services. Overall, service utilization increased. By project end, the 85% pentavalent-3 coverage target was achieved in Bambasi; Assosa fell short by 1%. Nonetheless, both were among the *woredas* that achieved >80% pentavalent-3 coverage in the 2015/16 Ethiopia Demographic and Health Survey; compared with a national average of 53%, [15, 16] and BGRS regional average of 76%. Similarly, the 85% target for measles coverage was achieved in Bambasi (86%), but not in Assosa (81%). This could be because Assosa is nearly twice as large as Bambasi and has more hard-to-reach areas. These rates still exceed the 80% district coverage recommended in the GVAP [17], the BGRS regional average (71%) and national average (54%) [15].

Basic HEP supportive activities implemented by the project allowed for more stable immunization delivery both at static posts and through outreaches, ultimately reaching more infants and reducing the burden of travel time and costs for families [11, 18]. Significant fluctuations in vaccine achievement and coverage were observed during the baseline period, showing stability and upward trend following implementation. However, in mid-2014, measles coverage in Bambasi declined, possibly due to vaccine stock-out, before the project introduced targeted basic EPI support to help assure vaccines were available and accessible to communities [9]. Capacity building of HEWs in interpersonal communication and consistent supportive supervision may have also contributed to increased utilization. Well-functioning health systems improve service availability and boost community confidence, possibly also increasing demand for services [7]. The project team therefore felt it necessary to bolster basic supply-side elements of EPI. Rollout of the HDA, within the HEP, likely also contributed to increased coverage. Further research is required to determine the extent to which project results are attributable to i) implementation of the cross-cutting community engagement approach and tools, in conjunction with HEP support, as compared to ii) basic HEP support from partners and iii) the HEP alone, without partner support.

Nearly 54% of all eligible infants in both *woredas* defaulted at least once during the project period. Involvement of caregivers in mining activities and associated travels were reported to the study team as one of the key causes for default [13]. Nonetheless, most defaulters (84%) were identified and subsequently caught up with vaccinations they had missed, exceeding the 70% target. There was no systematic immunization defaulter tracing strategy in place before the project. Therefore, it is probable that this result stems from the use of the DTT and HEW-community co-managed defaulter identification and follow-up.

Also important is the use of the *Enat Mastawesha*. The appointment reminder system may have contributed to reducing defaulters by creating a forum for health workers to provide MNCH education to mothers. Studies have shown fewer defaults and higher immunization completion when caregivers discussed immunization with HEWs [10] or other families in the community [19]. In addition, caregivers who were visited at home by HEWs have been shown to have better immunization completion rates [20]. Use of maternal health services has also been associated with completion of immunizations [7, 10]. The FCP promoted the use of ANC with the *Enat Mastawesha* and supported the EPI system. These activities likely contributed in a synergistic manner to improved immunization coverage.

The proportion of postpartum women adopting a modern FP method as well as the uptake of ANC increased. Project *woredas* performed higher than the national average for both one and four ANC visits. This may be due to early engagement with pregnant women and key household decision-makers during facilitated discussions using the *Enat Mastawesha* and increased capacity of the HEWs in interpersonal communication, counseling skills and implant insertion. Additionally, a recent study from Ethiopia identified data-driven decision-making and positive relationships between health facilities, HEWs, and the community as common underlying factors in high-performing *woredas* [21]. These factors were key components of the FCP, thus it is plausible to postulate that the intervention contributed to increased uptake of the services assessed.

### Limitations

This study had limitations. The analysis of routine data do not control for key confounders, including other health interventions being implemented or general health or socioeconomic improvements, that could have also contributed to improvements in key indicators reported here. The formative evaluation also did not include a control group. Thus, it is difficult to make a causal attribution to the intervention. The CORE Group Polio Project supporting polio campaigns and community-based surveillance for acute flaccid paralysis and other vaccine-preventable diseases in the same region may have affected the indicators reported; however, that program did not fully cover the two woredas. Similarly, during the project period, EngenderHealth supported health centers in the region to provide long acting reversible contraceptives; these activities may also have affected to the FP indicators reported. The Woreda Health Office provided forecasted infant populations based on the most recent census conducted in 2007, thus denominator estimates may not be accurate and may affect coverage. Also, the Regional Health Bureau reduced the conversion factor used to estimate the number of infants in a population. Thus, we observed a drop in the estimated number of infants due for vaccinations in 2015–2016 compared to 2013–2014. This drop could have contributed to the increases in immunization coverage. We also did not have a large enough sample size to produce stable monthly estimates. Finally, this study design did not allow us to conduct a cost-effectiveness analysis or to assess timeliness of service uptake.

## Conclusion

The FCP intervention has the potential to improve immunization uptake by promptly identifying defaulters and getting them caught up on missed vaccines as well as by improving the use of perinatal services including ANC and postpartum FP utilization. The immunization coverage rates observed may be due to the intensified involvement of community leaders, HEWs and HDAs in defaulter tracing. Improvement in the uptake of select perinatal services may be due to improved community and caregiver engagement approaches including the use of *Enat Mastawesha* as well as improvement in the technical capacity of HEWs. This study provides evidence to warrant further research to better determine the impact of such a strategy on sustained increases in immunization rates and other MNCH services. An impact evaluation, including a cost-benefit analysis of the approach, will be the next step in producing evidence of impact on service uptake.

## Supporting information

S1 Fig Theory of change. (DOCX)

S1 File (DO)

## References

[1] Ozawa S, Clark S, Portnoy A, Grewal S, Brenzel L, Walker DG. Return on Investment from Childhood Immunization In Low- And Middle-Income Countries, 2011–20. *Health Aff (Millwood)*. 2016;35(2):199–207. 10.1377/hlthaff.2015.1086 26858370

[2] WHO. *Immunization Coverage Factsheet (Reviewed March 2017)*.World Health Organization; 2017.

[3] WHO. *Estimates for child causes of death*, *2000–2016*. World Health Organization; 2017.

[4] Casey RM, Dumolard L, Danovaro C, et al *Global routine vaccination coverage*, *2015*. Geneva: World Health Organization; 2016. 10.15585/mmwr.mm6545a5 27855146

[5] *Ethiopia Demographic and Health Survey 2011*. Addis Ababa, Ethiopia and Calverton, Maryland, USA: Central Statistical Agency and ICF International; 2012.

[6] WHO. *2015 Assessment Report of the Global Vaccine Action Plan*. Geneva: World Health Organization; 2016.

[7] Tadesse H, Deribew A, Woldie M. Predictors of defaulting from completion of child immunization in south Ethiopia, May 2008: a case control study. *BMC Public Health*. 2009;9:150 10.1186/1471-2458-9-150 19463164 PMC2694177

[8] Etana B, Deressa W. Factors associated with complete immunization coverage in children aged 12–23 months in Ambo Woreda, Central Ethiopia. *BMC Public Health*. 2012;12:566 10.1186/1471-2458-12-566 22839418 PMC3508824

[9] Animaw W, Taye W, Merdekios B, Tilahun M, Ayele G. Expanded program of immunization coverage and associated factors among children age 12–23 months in Arba Minch town and Zuria District, Southern Ethiopia, 2013. *BMC Public Health*. 2014;14:464 10.1186/1471-2458-14-464 24884641 PMC4032449

[10] Tesfaye F, Tamiso A, BIrhan Y, Tadele T. Predictors of Immunization Defaulting among Children Age 12–23 Months in Hawassa Zuria District of Southern Ethiopia. *International Journal of Public Health Science (IJPHS)*. 2014;3(3):185–193.

[11] Legesse E, Dechasa W. An assessment of child immunization coverage and its determinants in Sinana District, Southeast Ethiopia. *BMC Pediatr*. 2015;15:31 10.1186/s12887-015-0345-4 25886255 PMC4438454

[12] *Fifth Child Project Interim Report*. Addis Ababa, Ethiopia: International Rescue Committee Ethiopia; 7 2017.

[13] Chantler T, Karafillakis E, Wodajo S, et al 'We All Work Together to Vaccinate the Child': A Formative Evaluation of a Community-Engagement Strategy Aimed at Closing the Immunization Gap in North-West Ethiopia. *Int J Environ Res Public Health*. 2018;15(4). 10.3390/ijerph15040667 29614056 PMC5923709

[14] Health Sector Transformation Plan (2015/16–2019/20). In. Addis Ababa, Ethiopia: Federal Ministry of Health; 2015.

[15] *Ethiopia Demographic and Health Survey 2015–16*: *Key Indicators*. Addis Ababa, Ethiopia and Calverton, Maryland, USA: Central Statistical Agency [Ethiopia] and ICF International;2017.

[16] Addressing the Gap: Equity and Quality in Health Care to Ensure Highest Possible Level of Health for All in Ethiopia Executive Summary on Key Achievements (2015/16). In: Federal Ministry of Health; 2016.

[17] WHO. *Global Vaccine Action Plan 2011–2020*. Geneva, Switzerland: World Health Organization;2013.

[18] Okwaraji YB, Mulholland K, Schellenberg JR, Andarge G, Admassu M, Edmond KM. The association between travel time to health facilities and childhood vaccine coverage in rural Ethiopia. A community based cross sectional study. *BMC Public Health*. 2012;12:476 10.1186/1471-2458-12-476 22726457 PMC3439329

[19] Lakew Y, Bekele A, Biadgilign S. Factors influencing full immunization coverage among 12–23 months of age children in Ethiopia: evidence from the national demographic and health survey in 2011. *BMC Public Health*. 2015;15:728 10.1186/s12889-015-2078-6 26224089 PMC4520202

[20] Mohamud AN, Feleke A, Worku W, Kifle M, Sharma HR. Immunization coverage of 12–23 months old children and associated factors in Jigjiga District, Somali National Regional State, Ethiopia. *BMC Public Health*. 2014;14:865 10.1186/1471-2458-14-865 25146502 PMC4158082

[21] Fetene N, Linnander E, Fekadu B, et al The Ethiopian Health Extension Program and Variation in Health Systems Performance: What Matters? *PLoS One*. 2016;11(5):e0156438 10.1371/journal.pone.0156438 27227972 PMC4882046

