# Supplementary figures and images for: Community engagement strategy for increased uptake of routine immunization and select perinatal services in north-west Ethiopia: A descriptive analysis
Source: PLoS One. 2020 Oct 29;15(10):e0237319. doi: 10.1371/journal.pone.0237319 (PMC7595373; doi:10.1371/journal.pone.0237319)

**
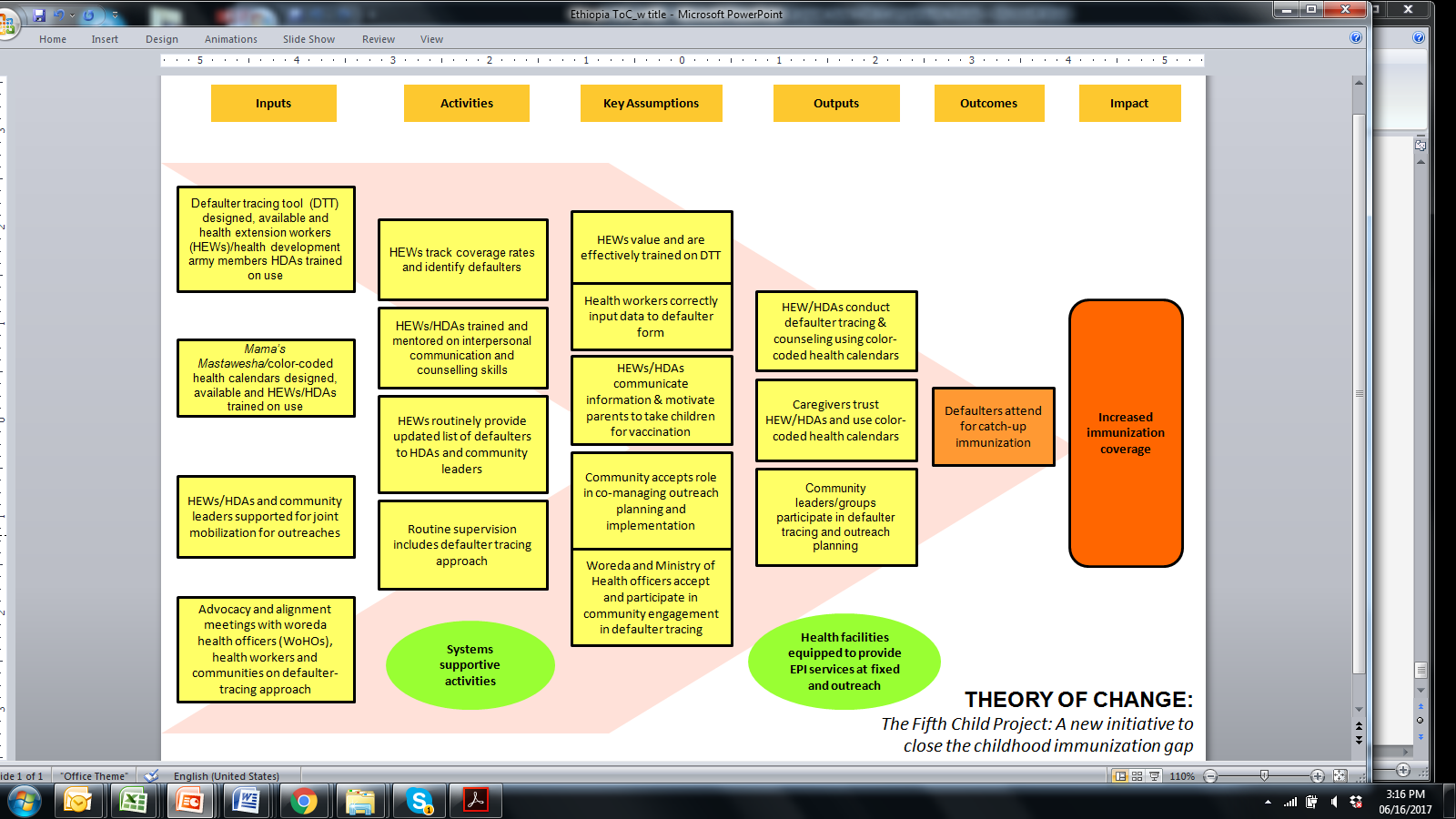
S1 Fig. Theory of Change**

Supplement: S1 Fig — (DOCX) [file pone.0237319.s001.docx]
